# Supplementary material for: Dark matter from axion strings with adaptive mesh refinement
Source: Nat Commun. 2022 Feb 25;13:1049. doi: 10.1038/s41467-022-28669-y (PMC8881528; doi:10.1038/s41467-022-28669-y)
Supplement: Supplementary file 1 — Supplementary Information [file 41467_2022_28669_MOESM1_ESM.pdf]

Supplementary Figures and Tables for Dark Matter from Axion Strings with Adaptive Mesh Refinement

Malte Buschmann, Joshua W. Foster, Anson Hook, Adam Peterson, Don E. Willcox, Weiqun Zhang, and Benjamin R. Safdi

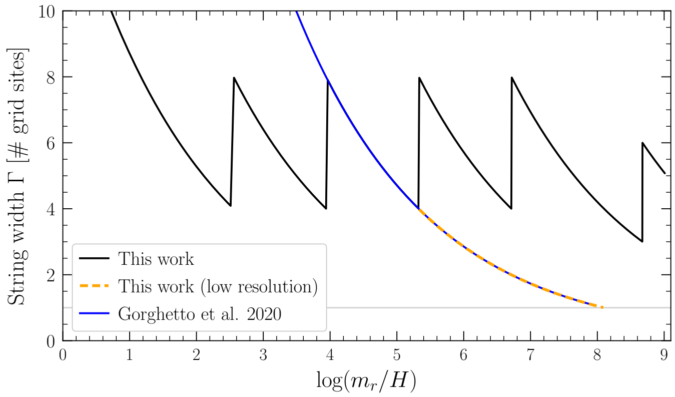

Supplementary Figure 1. **Evolution of the string width resolution.** String width Γ in units of number of grid sites as a function of simulation time parameterized by $\log m_r/H$. Each sudden increase in string width is due to the transformation $\Delta x \to \Delta x/2$ when an extra level is added. The first four refinement levels are added when $\Gamma/\Delta x = 4$ leading to a sudden increase to $\Gamma/\Delta x = 8$ each time. To test the effect of limited resolution we performed a low-resolution simulation (dashed red) that is identical to the main result (solid black) up to $\log m_r/H \approx 5.3$ but does not add any extra refinement levels afterwards. We compare this to the approximate resolution of simulations on a static lattice (blue) presented in [1]. Simulations on a static lattice over-resolve string cores at early times while under-resolving them at late times. The gray horizontal line corresponds to the often used resolution criteria $m_r \Delta x \lesssim 1$.

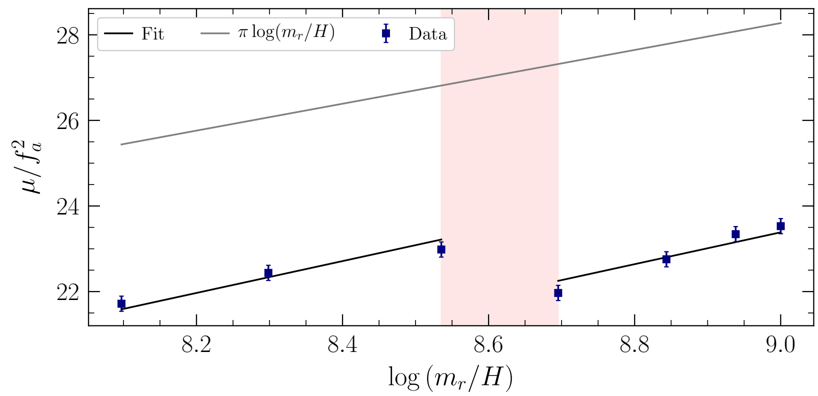

Supplementary Figure 2. **Evolution of the string tension.** The string tension realized in our simulation as a function of time (see Methods Sec. H for details of how this is computed). The theoretical string tension as a function of $\log m_r/H$ is shown in grey, to leading order in large log, while the string tensions measured in our simulation with data driven errors are shown in dark blue. Only the leading log growth of the data is expected to match the theoretical expectation; we find consistency between the linear growth in $\log m_r/H$ of the theoretical and measured string tensions. Note that an additional refinement level is added during the red band, at $\log m_r/H \approx 8.7$, leading to a change in the overall offset of the μ data. Error bars correspond to the normally distributed 68% confidence intervals.

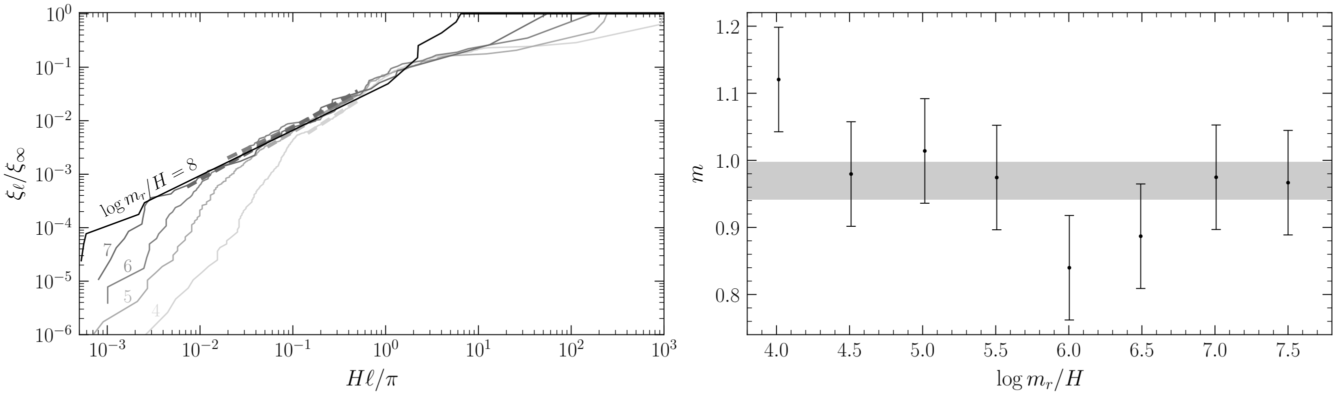

Supplementary Figure 3. **Distribution of string loop lengths.** (Left) The total string length in string loops smaller than ℓ, ξ_ℓ, versus ℓ for various values of $\log m_r/H$. It is clear that as time progresses the string loop distribution is approaching an attractor solution. We perform a power-law fit of the form $\xi_\ell = D\ell^m$ to the string loop distribution within the attractor regime (dashed lines). (Right) Distribution of the index m. The result is joint assuming no time dependence finding $m = 0.97 \pm 0.03$ (gray band). As argued in the text, this attractor solution leads to an axion spectrum with $q = 1$. Error bars correspond to the normally distributed 68% confidence intervals.

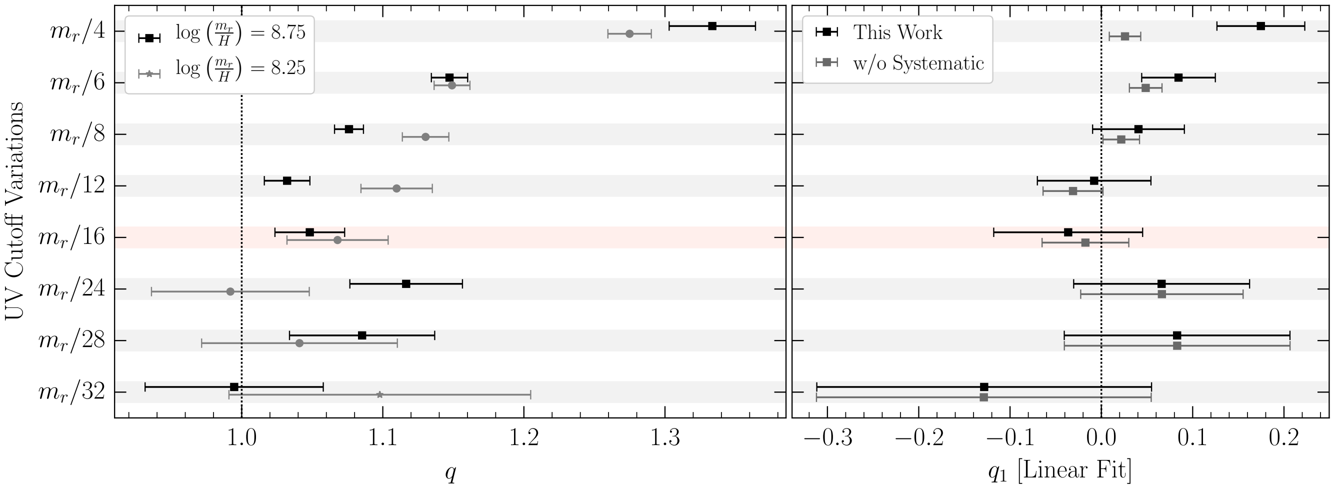

Supplementary Figure 4. **Fit results for the axion emission spectral evolution over time with varying UV cut-offs.** (Left) A comparison of the best-fit values for the index q of string emission at selected times for several choices of the UV cutoff of the fit range. (Right) Same as left but for q_1. All index evolution results for these variations are presented in detail in Tab. II. Note that the "w/o Systematic" data points do not include the systematic nuisance parameter σ as given in the likelihood in (25). Error bars correspond to the normally distributed 68% confidence intervals.

Coefficient	$x_{\rm IR} = 30$	$\mathbf{x_{IR} = 50}$	$x_{\rm IR} = 75$	$x_{\rm IR} = 100$
q_1	0.07 ± 0.07	$\mathbf{-0.04 \pm 0.08}$	-0.06 ± 0.13	-0.32 ± 0.26
q_0	0.41 ± 0.58	$\mathbf{1.36 \pm 0.69}$	1.5 ± 1.12	3.68 ± 2.21
$q_0^{\rm const.}$	0.98 ± 0.04	$\mathbf{1.02 \pm 0.04}$	1.0 ± 0.05	1.02 ± 0.07

Table I. **Fit results for the spectral evolution with different IR cutoffs.** Results of the fits to the spectral evolution holding all our fiducial analysis choices fixed but for various IR cutoffs $x_{\rm IR}$. We provide the fits and uncertainties for the q_1 and q_0 in the linearly growing index model and the best fit constant for $q_0^{\rm const.}$ in the constant index model. Our fiducial choice of $x_{\rm IR} = 50$ is shown in bold.

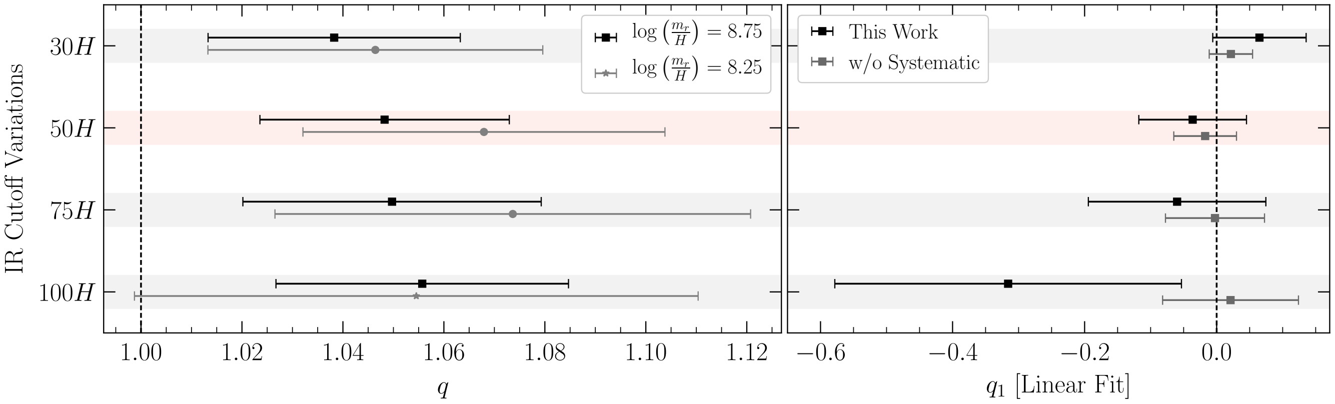

Supplementary Figure 5. **Fit results for the axion emission spectral evolution over time with varying IR cut-offs.** As in Fig. 4, but comparing choices of the IR cutoff. All index evolution results for these variations are presented in detail in Tab. I. Error bars correspond to the normally distributed 68% confidence intervals.

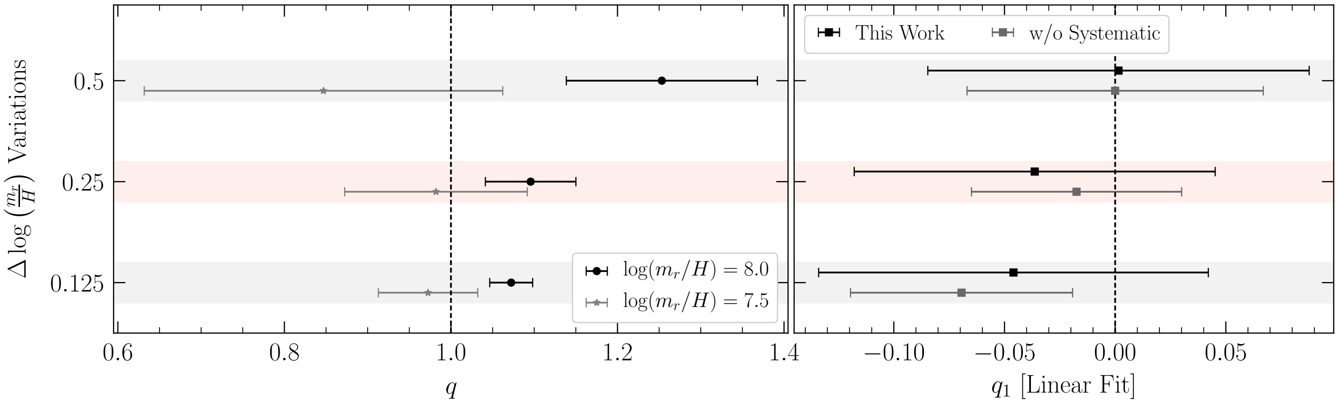

Supplementary Figure 6. **Fit results for the axion emission spectral evolution over time with varying $\Delta\log(m_r/H)$ choices.** As in Fig. 4, but comparing choices of $\Delta\log(m_r/H)$ used in calculating the instantaneous emission spectrum. All index evolution results for these variations are presented in detail in Tab. III. Error bars correspond to the normally distributed 68% confidence intervals.

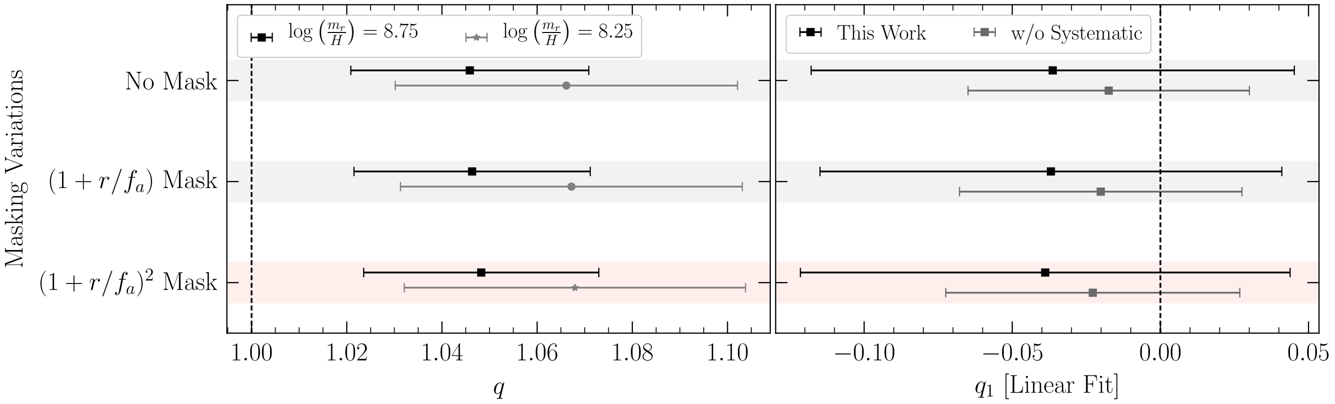

Supplementary Figure 7. **Fit results for the axion emission spectral evolution over time with varying masking functions.** As in Fig. 4, but comparing choices of the string masking function. All index evolution results for these variations are presented in detail in Tab. IV. Error bars correspond to the normally distributed 68% confidence intervals.

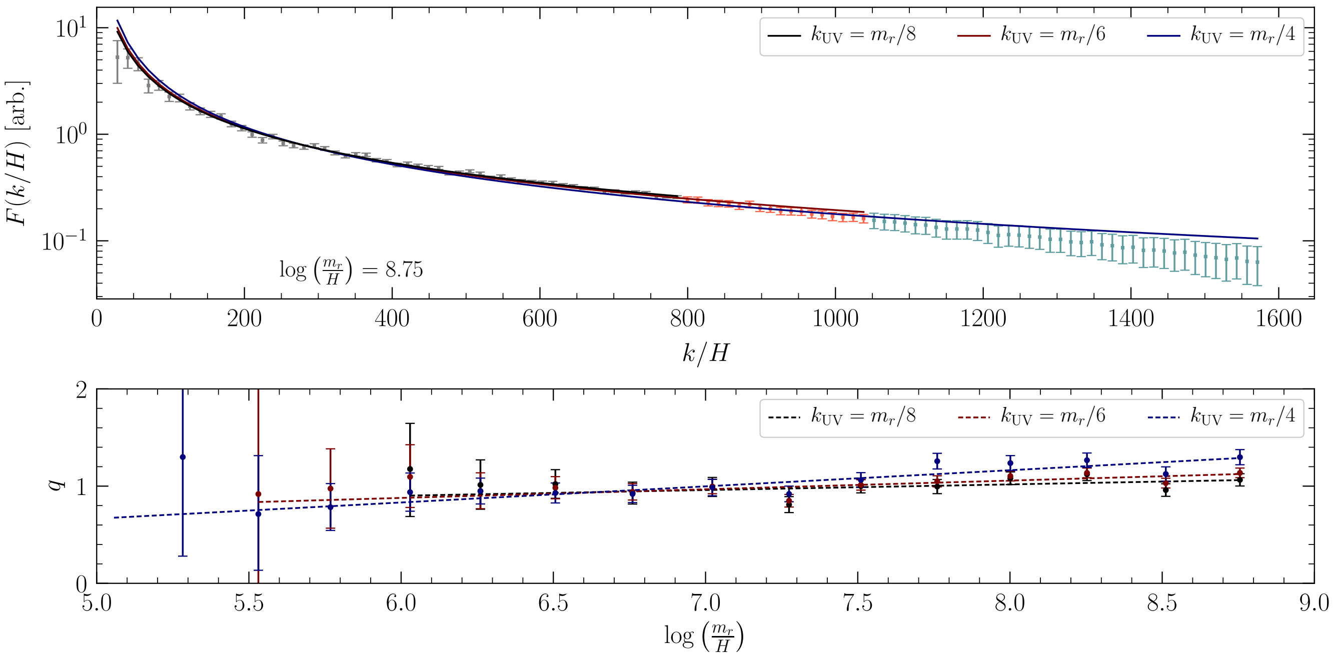

Supplementary Figure 8. **Systematic variations to the instantaneous emission spectrum fitting range.** (Top) Example fits to the instantaneous emission spectrum at $\log(m_r/H) = 8.75$ for our three largest choices of the UV cutoff for the fitting range. The data indicated in grey corresponds to the range $k \in (30H, m_r/8)$, with associated fit in black. In red, which show the fit obtained with the range $k \in (30H, m_r/6)$, which includes the grey and additionally light-red data. In dark blue, we show the fit obtained with the range $k \in (30H, m_r/4)$, which includes the grey, light-red, and light-blue data. Error bars have been obtained in a data-driven way from the fits using the procedure described in Methods Sec. I. Visible mismodeling at large k/H biases the fitted power-law towards artificially larger q. This can be contrasted with the results shown in Fig. 3, where a more conservative choice of UV cutoff does not result in apparent mismodeling at an identical time. (Bottom) The time evolution of the emission spectrum index for these large choices of UV cutoff for the fitting range. A clear trend of increasing q is obtained for the largest UV cutoff, suggesting that choices of large UV cutoff may result in unphysical growth in the fitted spectral index. Evidence for the linear growth of q in $\log(m_r/H)$ was claimed in [1] based on analysis performed with the fitting range $k \in (30H, m_r/4)$. Error bars correspond to the normally distributed 68% confidence intervals.

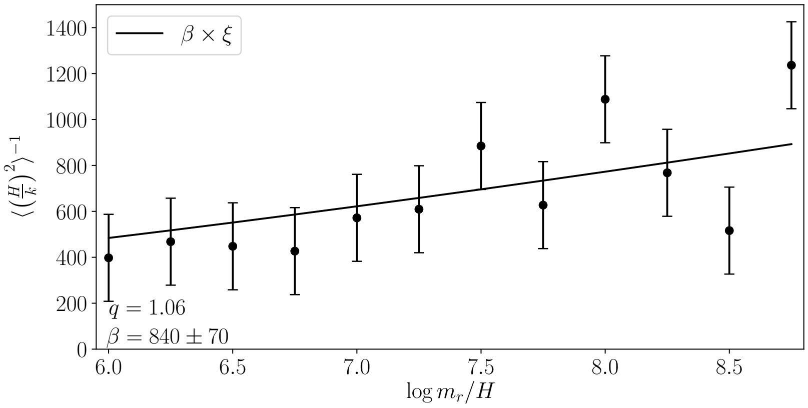

Supplementary Figure 9. **Evolution of the inverse axion momentum squared over time.** The inverse expectation value $\langle (H/k)^2 \rangle^{-1}$ computed using the axion spectrum $F(k/H)$ by numerically integrating the spectrum to $k/H = x_{\rm max} = 50$ and then analytically integrating the power law distribution $F(x) \propto x^{-q}$ from $x_{\rm max}$ to the UV cut-off at $k/H \sim e^{\log_*}$ for $\log_* \approx 65$ (as in Fig. 4). Smaller values of β correspond to larger axion field values. Here, we illustrate the result for the maximum allowed q of 1.06, which leads to the smallest β consistent with our simulation results. Error bars correspond to the normally distributed 68% confidence intervals.

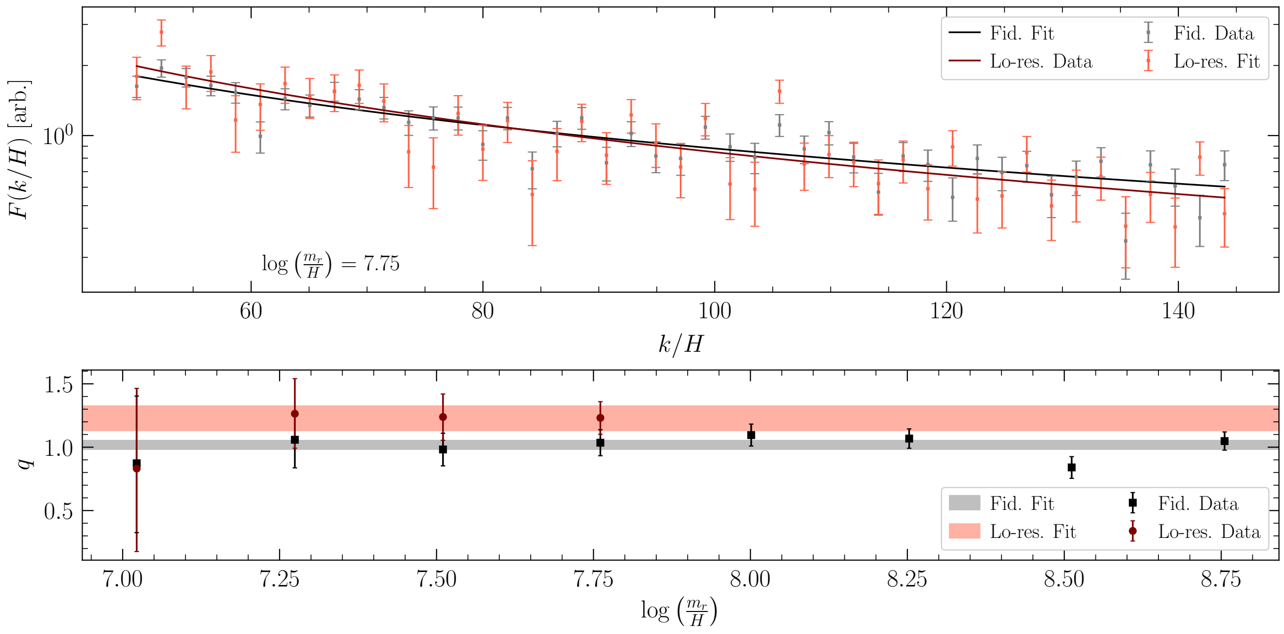

Supplementary Figure 10. **Dependence of the instantaneous emission spectrum on the simulation resolution.** As in Fig. 3, but comparing fits to the emission spectrum and index evolution for our fiducial simulation output and a lower-resolution simulation. The two simulations are identical until $\log m_r/H \approx 5.3$ when the low-resolution simulation stops adding extra refinement levels. The low-resolution simulation is then run until $\log m_r/H \approx 8$, when we saturate the $m_r \Delta x \lesssim 1$ resolution requirement. (Top) A comparison of the emission spectra and fits for the fiducial simulation (data in grey, fit in black) and the lower-resolution simulation (data in light red, fit in maroon) at $\log m_r/H \approx 7.75$, which is the final emission spectrum obtained in our lower-resolution simulation. The lower-resolution simulation prefers a larger power-law index in the fit, and the data-driven errors are somewhat larger than in our fiducial simulation. (Bottom) A comparison of the best-fit emission spectrum index as a function of $\log m_r/H$ for the fiducial and lower-resolution simulation. Over the range of $\log m_r/H$ to which we are sensitive in the lower-resolution simulation, the emission spectra realize larger indices, suggesting that resolution loss in a uniform resolution simulation that saturates the resolution criteria may lead to a systematic bias towards index growth. Error bars correspond to the normally distributed 68% confidence intervals.

Coefficient	$x_{\rm UV} = 4$	$x_{\rm UV} = 6$	$x_{\rm UV} = 8$	$x_{UV} = 12$	$\mathbf{x_{UV} = 16}$	$x_{\rm UV} = 24$	$x_{\rm UV} = 28$	$x_{\rm UV} = 32$
q_1	0.17 ± 0.05	0.08 ± 0.04	0.04 ± 0.05	-0.01 ± 0.06	$\mathbf{-0.04 \pm 0.08}$	0.08 ± 0.09	0.08 ± 0.12	-0.2 ± 0.2
q_0	-0.22 ± 0.37	0.39 ± 0.32	0.7 ± 0.41	1.09 ± 0.51	$\mathbf{1.36 \pm 0.69}$	0.36 ± 0.78	0.34 ± 1.05	2.74 ± 1.68
$q_0^{\rm const.}$	1.12 ± 0.05	1.06 ± 0.03	1.03 ± 0.03	1.03 ± 0.03	$\mathbf{1.02 \pm 0.04}$	1.05 ± 0.04	1.05 ± 0.04	1.03 ± 0.05

Table II. **Fit results for the spectral evolution with different UV cutoffs.** As in Tab. I, but for varying UV cutoff $x_{\rm UV}$ with all other parameters fixed to their fiducial values. Our fiducial choice of $x_{\rm UV} = 16$ is shown in bold.

Coefficient	$\Delta \log = 0.125$	$\mathbf{\Delta log = 0.25}$	$\Delta \log = 0.5$	$\Delta \log = \log 2$
q_1	0.0 ± 0.09	$\mathbf{-0.04 \pm 0.08}$	-0.05 ± 0.09	-0.1 ± 0.06
q_0	1.02 ± 0.72	$\mathbf{1.36 \pm 0.69}$	1.4 ± 0.73	1.84 ± 0.46
$q_0^{\rm const.}$	1.03 ± 0.04	$\mathbf{1.02 \pm 0.04}$	1.02 ± 0.03	1.03 ± 0.01

Table III. **Fit results for the spectral evolution with different steps in $\log(m_r/H)$.** As in Tab. I, but now holding all our fiducial analysis choices fixed, with the exception of the size of the step in $\log(m_r/H)$ used in the finite difference for the calculation of the instantaneous emission spectrum. We vary between $\Delta \log(m_r/H) \in \{0.125, .25, .5, \log(2)\}$, with the $\log(2)$ differences corresponding to a Hubble time spacing. Our fiducial choice of $\Delta \log(m_r/H) = 0.25$ is shown in bold.

Coefficient	**Eq. 9 Mask**	Eq. 10 Mask	Eq. 11 Mask
q_1	$\mathbf{-0.04 \pm 0.08}$	-0.04 ± 0.08	-0.05 ± 0.08
q_0	$\mathbf{1.36 \pm 0.69}$	1.37 ± 0.65	1.39 ± 0.7
$q_0^{\text{const.}}$	$\mathbf{1.02 \pm 0.04}$	1.02 ± 0.03	1.02 ± 0.03

Table IV. **Fit results for the spectral evolution with different string masks.** As in Tab. I, but now holding all our fiducial analysis choices fixed, with the exception of the choice of screening mask. We vary this choice between the screening functions described in (9), (10), and (11). Our fiducial choice of screening in the form of (9) is shown in bold.

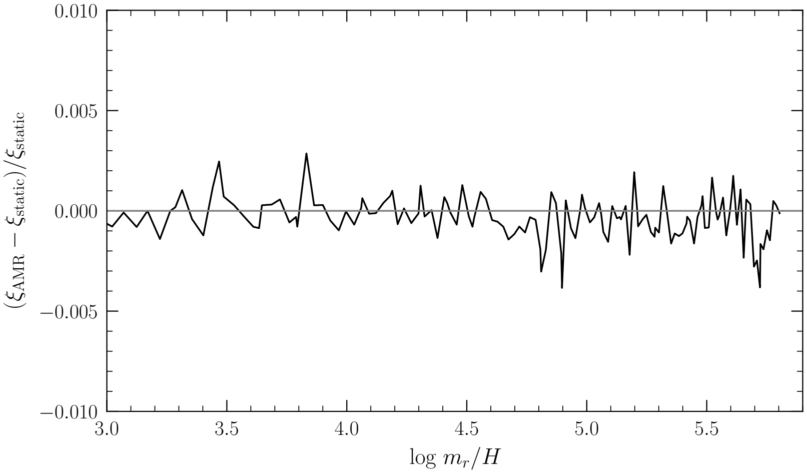

Supplementary Figure 11. **The difference in string lengths per Hubble with and without the AMR framework.** Relative difference between string lengths computed from a simulation with a high-resolution static grid, ξ_{static}, and an AMR grid, ξ_{AMR}, using identical initial states. The difference between both simulations is less than 0.4% and centered around zero with no observable drift. This provides evidence that the AMR method yields compatible results with the static grid method. The advantage of AMR, however, is that it is easier to simulate to larger log values.

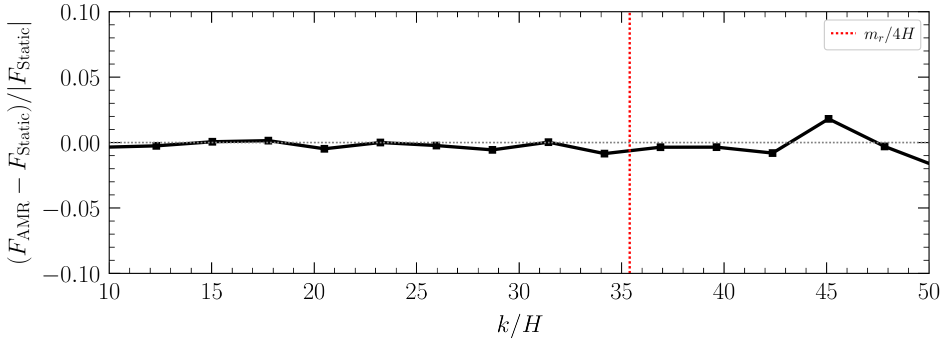

Supplementary Figure 12. **The difference in the instantaneous spectrum with and without the AMR framework.** As in Fig. 11, but for the instantaneous axion emission spectrum F as a function of k/H. The spectrum is computed by comparing the states between $\log m_r/H = 4.953$ and $\log m_r/H = 5.790$. The largest k considered in this work, relative to m_r, is $k = m_r/4$, which is indicated in vertical dashed red. Below the $k/H = m_r/4H$, the precision-limited differences in the emission spectra are below the one-percent level and thus subdominant compared to our statistical uncertainties.

$L/k_{\max}$	1.8 (1)	1.8 (2)	1.8 (Stacked)	8.8 (1)	8.8 (2)	8.8 (Stacked)	4.8 (Fiducial)
q	0.91 ± 0.37	0.98 ± 0.18	0.98 ± 0.28	0.96 ± 0.21	1.05 ± 0.15	1.00 ± 0.15	0.92 ± 0.09

Table V. **Dependence of the axion radiation spectral index on the initial simulation state.** Tabulated results of the spectral index q for the different statistical realizations of the simulations using different initial mode numbers, as described in Methods Sec. K. We compare the best-fit power-law indices at $\log = 6.75$ for each simulation at $L/k_{\max} = 1.8$ and $L/k_{\max} = 8.8$ and their stacked results with our fiducial simulation using $L/k_{\max} = 4.8$, which all demonstrate mutual compatibility.

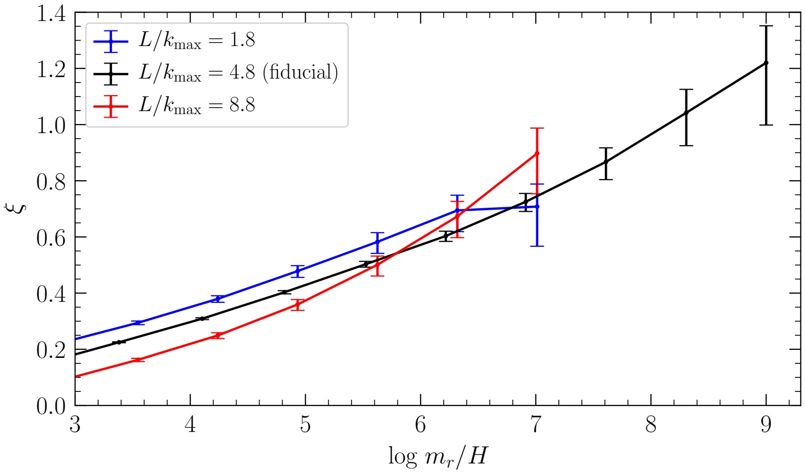

Supplementary Figure 13. **Dependence of the number of strings per Hubble on the initial state.** The string length per Hubble volume ξ for various different initial states and simulation setups. $L/k_{\max}$ is a measure for the physical size of the smallest mode included in the initial state and sets the initial string density. Shown in black is our main simulation result. The red and blue curves represent simulations with a different $L/k_{\max}$ ratio in the initial state. Note that we have averaged the results of the two statistical realizations of each for illustration. These results support the hypothesis that independent of the initial string density all simulations appear to converge to a common ξ scaling with $\log m_r/H$ after the PQ phase transition. Error bars correspond to the normally distributed 68% confidence intervals.

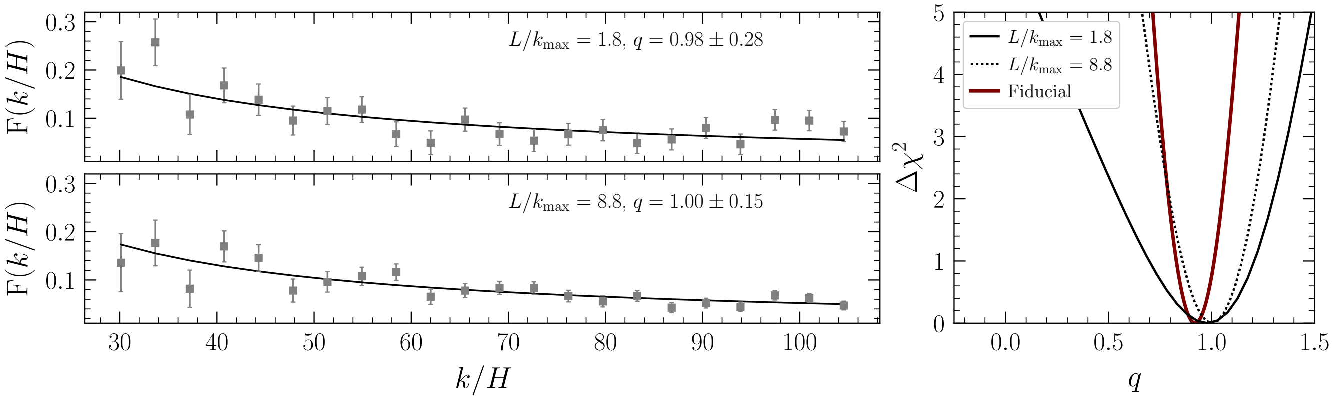

Supplementary Figure 14. **Dependence of the instantaneous spectrum on the number of initial mode numbers.** A comparison of axion emission spectra for various different initial states and simulation setups. (Top Left) The emission spectrum data (grey) and best-fit power-law model (black) for the average of two simulations performed with $L/k_{\max} = 1.8$ at $\log = 6.75$. (Bottom Left) The emission spectrum data (grey) and best-fit power-law model (black) for the average of two simulations performed with $L/k_{\max} = 8.8$ at $\log = 6.75$. (Right) A comparison of the likelihood profiles for the power-law index for the two systematic tests of the initial state, averaged over both the two statistical ensembles. We illustrate the likelihood profiles for the two different initial mode number cases along with our fiducial simulation result (red). Error bars correspond to the normally distributed 68% confidence intervals.

[1] Marco Gorghetto, Edward Hardy, and Giovanni Villadoro, "More Axions from Strings," SciPost Phys. **10**, 050 (2021), arXiv:2007.04990 [hep-ph].
